# Suspension of basal insulin to avoid hypoglycemia in type 1 diabetes treated with insulin pump

**DOI:** 10.1530/EDM-14-0081

**Published:** 2015-01-01

**Authors:** Mauro Boronat, Rosa M Sánchez-Hernández, Julia Rodríguez-Cordero, Angelines Jiménez-Ortega, Francisco J Nóvoa

**Affiliations:** 1 Section of Endocrinology and Nutrition, Hospital Universitario Insular, Las Palmas de Gran Canaria, 35016, Spain; 2 Department of Medical and Surgical Sciences, University of Las Palmas de Gran Canaria, Las Palmas de Gran Canaria, Spain

## Abstract

Treatment with continuous s.c. insulin infusion (CSII) provides better glycemic control and lower risk of hypoglycemia than conventional therapy with multiple daily insulin injections. These benefits have been related to a more reliable absorption and an improved pharmacokinetic profile of insulin delivered through CSII therapy. However, even for patients treated with CSII, exaggerated postmeal hyperglycemic excursions and late postabsorptive hypoglycemia can still constitute a therapeutic challenge. Two female patients with type 1 diabetes who began treatment with CSII required to increase their previous breakfast insulin-to-carbohydrate ratio in order to achieve postprandial glycemic goals. However, they simultaneously presented recurrent episodes of late hypoglycemia several hours after breakfast bolus. Advancing the timing of the bolus was ineffective and bothersome for patients. In both cases, the best therapeutic option was to set a basal insulin rate of zero units per hour during 6 h after breakfast. Even so, they need to routinely take a midmorning snack with 10–20 g of carbohydrates to avoid late postabsorptive hypoglycemia. They have been using this insulin schedule for about 3 years without complications. The action of prandial insulin delivered through insulin pumps can be inappropriately delayed for the requirements of some patients. Although suspension of basal rate can be an acceptable therapeutic alternative for them, these cases demonstrate that new strategies to improve the bioavailability of prandial insulin infused through CSII are still needed.

**Learning points:**

- CSII remains the most physiologically suitable system of insulin delivery available today.
- Additionally, the duration of action of prandial insulin delivered through insulin pumps can be excessively prolonged in some patients with type 1 diabetes.
- These patients can present recurrent late episodes of hypoglycemia several hours after the administration of insulin boluses.
- The routine suspension of basal insulin for several hours, leaving meal bolus to cover both prandial and basal insulin requirements, can be a therapeutic option for these subjects.

## Background

Treatment with continuous s.c. insulin infusion (CSII) provides better glycemic control for patients with type 1 diabetes mellitus than conventional therapy with multiple daily insulin injections, with a lower frequency of hypoglycemia and lesser insulin doses (1). Apart from the option to program customized flexible basal insulin infusion rates, superiority of CSII is derived from a better bioavailability of insulin, due to the smaller s.c. depot of the drug and the lower coefficient of variation of basal insulin absorption (2). In addition, insulin pumps incorporate automated bolus calculators, which permit to tailor insulin dose of prandial boluses according to glucose levels, glucose objectives, dietary carbohydrate quantity, insulin sensitivity, and estimated amount of insulin onboard.

In spite of these advantages, it is still possible that insulin delivered through CSII does not optimally fulfill the particular needs of some individuals. Herein, we present two patients with type 1 diabetes treated with CSII in whom peak action of breakfast insulin boluses is too delayed to achieve adequate postprandial glucose control but causes late episodes of hypoglycemia.

## Case presentation

Subjects are two female patients who regularly attended our Endocrinology Department and began CSII therapy in February 2011.

Case 1 had been diagnosed with type 1 diabetes by her private endocrinologist when she was 43 years old. She has been treated with a basal-bolus insulin regimen and was referred to our center at the age of 48 because of recurrent unpredictable episodes of hypoglycemia. Case 2 was diagnosed with gestational diabetes in her first pregnancy at the age of 27. She was then managed by her obstetrician with dietary and exercise recommendations and gave birth to a normal-weight, healthy baby. However, 6 months after delivery, she presented with symptoms of hyperglycemia. Laboratory investigations showed fasting plasma glucose of 232 mg/dl, HbA1c 12.9%, and glutamic acid decarboxylase antibodies 5 U/ml (normal value <1 U/ml). She also began with a basal-bolus insulin regimen, but despite adherence to multiple daily insulin injections with appropriate dose adjustments and frequent home blood glucose monitoring, glycemic control was suboptimal for the past few years. The main clinical features of both patients at the initiation of CSII are summarized in Table 1.

**Table 1:** Clinical characteristics of patients at pump placement and CSII settings after final dose adjustments

	**Case 1**	**Case 2**
Clinical features at CSII initiation		
Age (years)	50	43
BMI (kg/m^2^)	24.8	26.7
Diabetes duration (years)	7	14
Chronic complications of diabetes	None	None
Indication for CSII	Recurrent hypoglycemia	Elevated HbA1c
Treatment at the end of follow-up		
Total insulin dose (U/kg)	0.6	0.8
% Basal	26.4	32.1
% Bolus	73.6	67.9
Insulin-to-carbohydrate ratio (U/10 g carbohydrates)		
Breakfast	1.9	3.5
Lunch	1.4	2.7
Dinner	1.4	2
Sensitivity index	50	32
Basal rate (U/h)		
0000–0300 h	0.2	0.2
0300–0600 h	0.8	1.3
0600–0900 h	0.75	1
0900–1500 h	0	0
1500–1800 h	0.3	0.6
1800–2100 h	0.75	1.2
2100–2400 h	0.45	0.9
Mean HbA1c since CSII (%)	6.8±0.4	7.9±0.4

According to our usual practice, patients previously received a 1-week structured course of insulin pump therapy. They were both treated with a Minimed Paradigm Veo 754 pump (Medtronic Diabetes, Northridge, CA, USA), with lispro insulin. The total basal dose of insulin was calculated by decreasing 20% the previous amount of long-acting insulin (glargine in both cases), and therapy was begun with only one initial basal rate. Insulin requirements for preprandial boluses were based on insulin-to-carbohydrate ratio used for each meal during the weeks before pump placement. Therapy was modified at each follow-up visit according to capillary glucose measurements (premeals, 2 h after meals, and at bedtime). Specifically, basal rates were modified in order to keep fasting and daytime preprandial glucose levels between 70 and 130 mg/dl and bedtime glucose levels between 110 and 135 mg/dl. Bolus ratios were adjusted to achieve postprandial glucose levels lower than 180 mg/dl. Patients were trained and encouraged on the routine use of the automated bolus calculator feature of the pump (‘Bolus Wizard’), which was programed with the following settings: active insulin time 6 h, and blood glucose targets 80–120 mg/dl for preprandial time, 130–160 mg/dl for postprandial time, and 110–135 mg/dl for bedtime.

Basal rate adjustment through follow-up visits permitted the achievement of fasting glucose goals in both patients. However, it was noteworthy that postprandial glucose readings 2 h after breakfast were consistently above target. This led to a gradual increase in the breakfast insulin-to-carbohydrate ratio. However, in parallel with this measure, patients noted increasing episodes of hypoglycemia in the late morning time, between 3 and 5 h following breakfast. This problem was limited to breakfast. Postprandial glycemic excursions were adequately covered by lunch and dinner bolus without subsequent late hypoglycemia.

## Investigation

None of the patients presented lipohypertrophy or local allergic reactions on areas of catheter insertion. They denied any recent change to their customary breakfast. In case 1, breakfast was usually composed of a serving of milk and 20 g of ‘gofio’ (a traditional food in the Canary Islands, made from one or more roasted and ground cereals, mainly corn and/or wheat). Case 2 used to take a serving of milk and a ham sandwich (60 g of white bread). Patients were counseled to deliver breakfast bolus 30–60 min before eating, but apart from being bothersome for them, this recommendation failed to effectively prevent late hypoglycemia.

## Treatment

**Figure 1:**
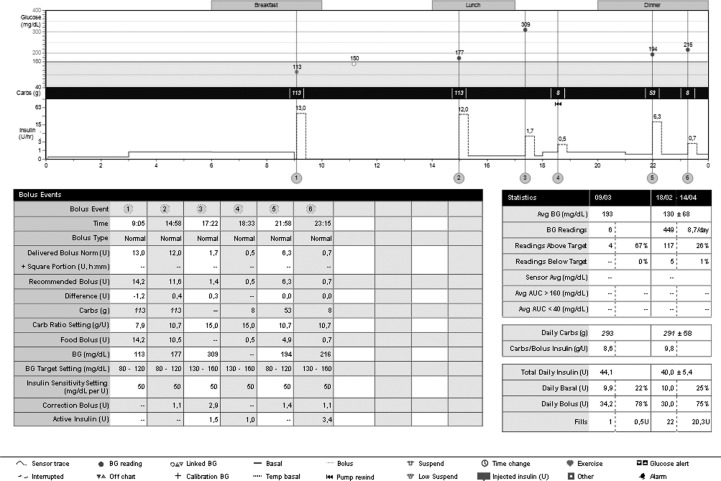
Example of a single day summary report of case 1, downloaded using the Medtronic CareLink software. On this weekend day, the patient ate an usually great amount of carbohydrates at breakfast. In the absence of insulin basal infusion, breakfast bolus achieved adequate postprandial glucose levels and maintained glucose levels until lunch. The patient took a not recorded midmorning snack.

Basal rate was progressively decreased during the hours following breakfast. Four months after beginning CSII therapy, both patients had set a basal rate of 0 units per hour from 0900 to 1500 h, i.e., from around 1 h after the breakfast bolus to 1–2 h after the lunch bolus. Even so, they still had to take a midmorning snack containing 10–20 g of carbohydrates to avoid hypoglycemia before lunch. A representative summary daily report of one of the patients, downloaded from the insulin pump data management software, is shown in Fig. 1.

## Outcome and follow-up

This insulin schedule has been satisfactorily used by patients for about 3 years, and no specific complications, such as frequent catheter clogging or severe hyperglycemia, have occurred. Insulin settings of CSII at the end of follow-up of both patients are shown in Table 1.

## Discussion

Achievement of postprandial glycemic control remains as one of the most challenging goals in the management of patients with type 1 diabetes. Modern devices for CSII incorporate several bolus strategies to account for differences in nutritional content of meals (standard, square, or dual-wave bolus), as well as bolus calculators intended to help patients in selecting insulin dose. However, as noted by other authors, these tools are still based on empiric measures and depend on the skills and experience of both the patient and the health professionals (3) (4).

In addition, postprandial glucose response shows a very high inter- and intra-subject variability, even when CSII is implemented under controlled experimental conditions (4). The poor reproducibility of insulin absorption is probably the most plausible cause for this high variability. In fact, it is obvious that pharmacokinetic profiles of prandial insulin boluses are unsatisfactory. By using euglycemic clamp techniques, it has been demonstrated that the action peak of a rapid-acting insulin analog delivered through an insulin pump occurs 90 min after the administration of a standard bolus, and the duration of action of the insulin extends up to 5 h (5). This finding is in accordance with the observation that insulin boluses result in significantly better postprandial glucose control when administered 15 min before a meal than when they are given just before the meal (6). Apart from the timing of infusion, pharmacokinetic properties of s.c. insulin infused through insulin pumps might be affected by other not well-studied factors, such as the duration of the catheter insertion, the site and depth of insertion, and the insulin depot around the tip of the catheter (7).

On the whole, although it is accepted that CSII therapy can be still defective for adequate control of postprandial hyperglycemia, the implications of these deficiencies on clinical practice have not merited sufficient attention and probably remain under-reported. In fact, to our knowledge, cases like the ones reported herein have not been described previously in the literature. In both cases, the action of insulin boluses was too slow with regard to the requirements of patients, leading to the appearance of late worrisome hypoglycemia. This problem was uniquely related to the breakfast bolus, probably because the insulin-to-carbohydrate ratio tends to be higher at breakfast, and basal insulin needs tend to be lower during the morning time (8). Stretching the interval time between bolus delivery and breakfast was ineffective, and it was inconvenient for patients, as they had to anticipate their normal waking time. Similarly, they did not also accept to indefinitely change their usual food choices for breakfast. Thus, therapeutic intervention mostly consisted of insulin therapy adjustment according to home glucose readings. After a progressive diminution, the basal rate was finally reduced to zero since 0900 h (∼1 h after breakfast) to 1500 h in both patients. Even so, patients still need to take a midmorning snack to prevent hypoglycemia before lunch.

The current approach for our patients is similar to, although more radical than, the super bolus strategy proposed by Walsh & Roberts (9), which consists of temporarily decreasing or stopping basal infusion, shifting this insulin to be delivered instead as additional part of a bolus. The super bolus was aimed to prevent postprandial hyperglycemia, while the simultaneous decrement in basal infusion should avoid late postabsorptive hypoglycemia. However, while the super bolus was mainly conceived to be used occasionally, for example, to cover meals with a high glycemic index, our patients have to interrupt basal insulin infusion for 6 h every day.

Studies performed on *in silico* subjects created from mathematical models for prediction of glucose concentrations in subjects with type 1 diabetes have suggested that the super bolus, or other strategies of combining basal and bolus insulin, could be habitually used instead of conventional bolus therapy, as they provide more effective control of postprandial hyperglycemia without increasing the risk of postabsorptive hypoglycemia (3). However, preliminary data on the application of these innovative algorithms for the infusion of prandial insulin *in vivo* seem to indicate that their theoretical advantages are limited by the unpredictability of individual glycemic responses (4). Anyway, it is possible that the future implementation of these more sophisticated methods to deliver prandial insulin can be useful for treatment of patients like those reported herein.

To date, daily prolonged suspension of basal infusion has been found to be safe for our patients. None of them has shown an increased trend toward catheter occlusion or unexplained hyperglycemia. In this regard, they have been instructed to set a temporary basal rate if they skip or delay breakfast.

In conclusion, the bioavailability of insulin infused through pumps for CSII does not fit to the needs of some particular patients. Improved algorithms for delivering prandial insulin or the introduction of novel faster insulin molecules could be future alternatives to overcome these drawbacks.

## Patient consent

Written informed consent was obtained from patients for publication of this article and the accompanying image.

## Author contribution statement

M Boronat and R M Sánchez-Hernández are the physicians of patients. M Boronat wrote the manuscript. J Rodríguez-Cordero and A Jiménez-Ortega are the nurses in the diabetes department who trained patients. F J Nóvoa reviewed the manuscript and contributed to the discussion.
